# Supplementary material for: Cell Therapy with Human Reprogrammed CD8+ T-Cells Has Antimetastatic Effects on Lewis Lung Carcinoma in C57BL/6 Mice
Source: Int J Mol Sci. 2022 Dec 12;23(24):15780. doi: 10.3390/ijms232415780 (PMC9779156; doi:10.3390/ijms232415780)
Supplement: Supplementary file 1 [file ijms-23-15780-s001.zip › ijms-2042041-supplementary.pdf]

Table S1. The effect of cell therapy with human reprogrammed $CD8^+$ T-cells isolated from blood of the healthy donor (human) on the content of lymphocytes (% of all stained mononuclear cells) in the blood of mice in a metastatic model of Lewis lung carcinoma on the 17th day of the experiment (M±m).

Immunophenotype/ Name of population	Intact control	Mice with LLC	Mice with LLC + hrT-cells
$CD8^+CD62L^{hi}CD197^{hi}CD95^-$/ Naive $CD8^+$ T-cells [33,34]	0	0	0.0001±0.00005
$CD8^+CD62L^+CD197^+CD95^{hi}$/ CD8 Memory T-cells ($T_{CM}$)	0.031±0.003	0.022±0.002[1]	0.019±0.009
$CD8^+CD62L^-CD197^-CD95^{hi}$/ Effector memory T-cells (T_{EMs}) [33,34,50]	0.018±0.002	0.011±0.001[1]	0.012±0.002
$CD8^+CD62L^-CD44^+$/ Highly differentiated effector memory T-lymphocytes [31]	0	0	0
$CD8^+CD44^{low}CD62L^{hi}$/ Naïve $CD8^+$ T-cells [33]	0.020±0.003	0.004±0.0003[1]	0.003±0.0003[1]
$CD8^+CD44^{hi}CD62L^{low}$ [33]	0	0	0
$CD8^+CD197^-$/ General population of non-naïve $CD8^+$ T-cells [51]	0.839±0.079	0.251±0.026[1]	0.312±0.032[1]

[1] — differences are significant in comparison with the intact control ($p<0.05$). Note: results are from 3 independent series of experiments.

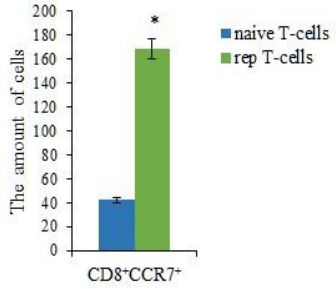

(a)

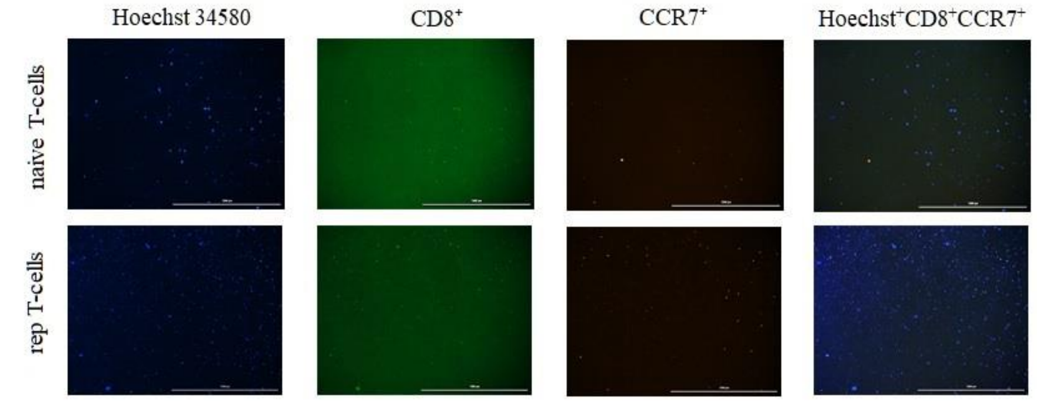

(b)

Figure S1. *In vitro* study of CCR7 (CD197) marker expression by naive and human reprogrammed $CD8^{+}$ T-cells isolated from the blood of healthy volunteer. (**a**) The count of naive and reprogrammed CD8+ T-cells isolated from the blood of healthy volunteer expressing the CCR7 marker in T-cell culture; (**b**) 4× images of T-cell stained with: Hoechst (blue) to identify cell nuclei; CD8 FITC (green); CCR7 AF555 (red); (Hoechst+CD8+CCR7+) composite image using all three colors. Determination of the percentage of cells CD8+CCR7+ is made by the ratio of cells counted in green and red channel to total cells counted using blue (DAPI) channel. All scale bars are 1000 μm. * – for comparison with the naive T-cells ($p<0.05$).

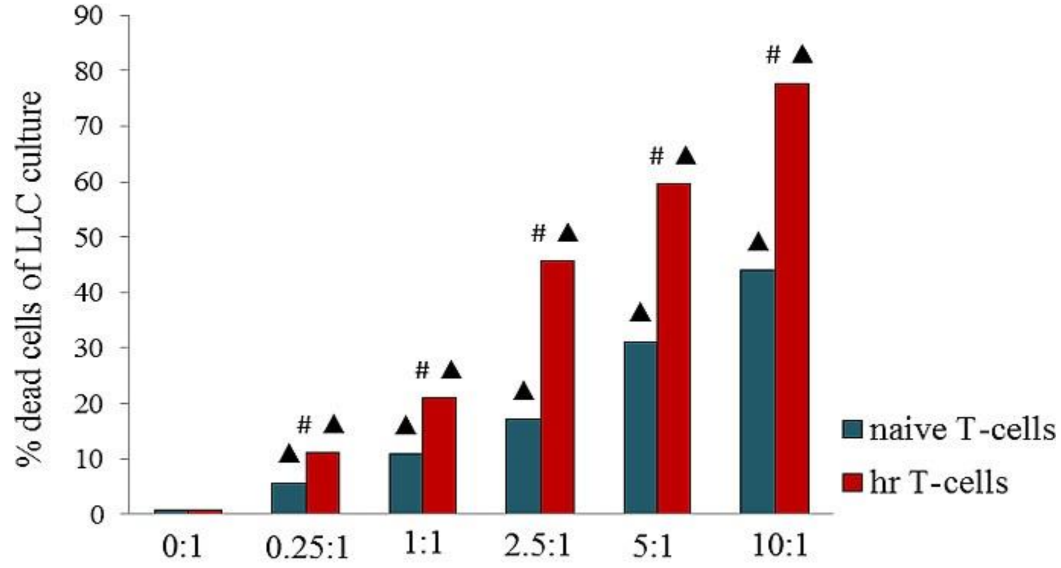

(a)

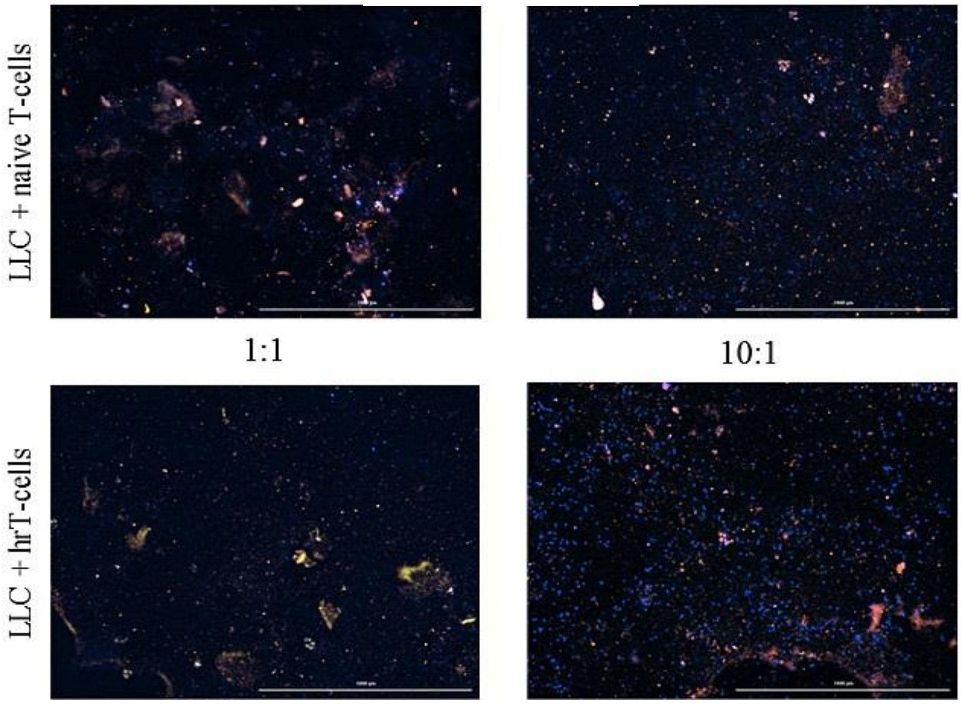

(b)

Figure S2. Cytotoxicity of human naive and reprogrammed CD8$^+$ T-cells isolated from the blood of healthy volunteer in LLC culture. **(a)** The count of apoptotic tumor LLC cells after co-cultivation with human naive or reprogrammed CD8$^+$ T-cells (% from dead cells of LLC culture); **(b)** Hoechst (blue) to identify cell nuclei; 7AAD (red); (Hoechst$^+$7AAD$^+$) composite image using all two colors. Determination of the percent of died cells of LLC Hoechst+7AAD+ is made by the ratio of cells counted in blue and red channel to total cells of LLC without green channel. All scale bars are 1000 µm. ▲ - for comparison with LLC control by Mann–Whitney test ($p < 0.05$); #- for comparison with the group "naive CD8$^+$ T-cell+tumor cells from LLC" by Mann–Whitney test ($p < 0.05$).

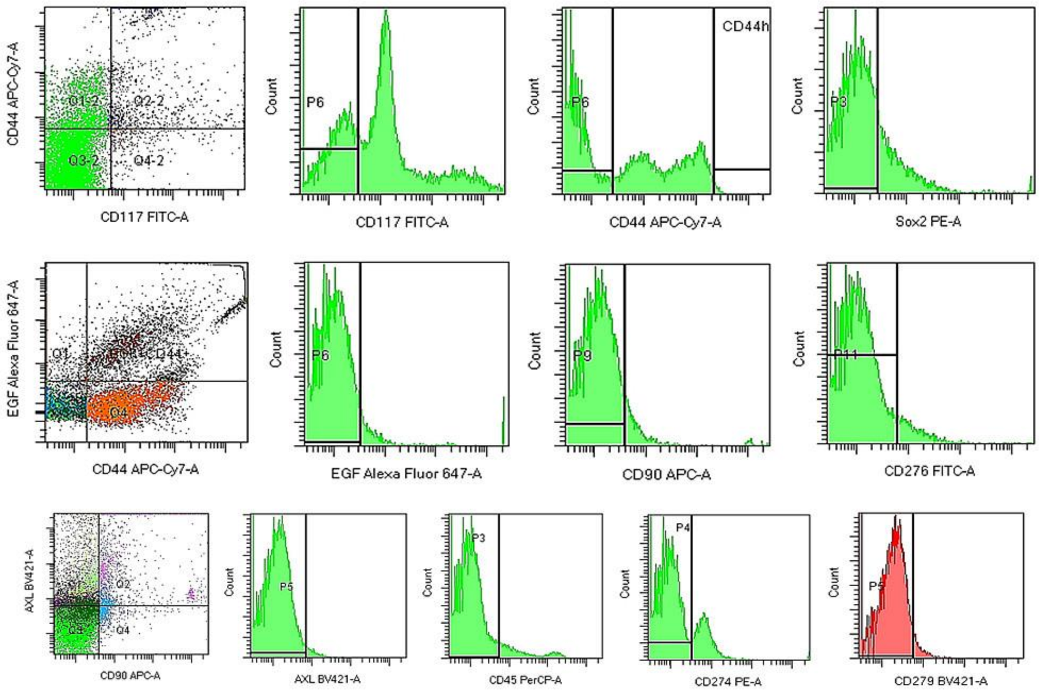

Figure S3. The effect of therapy with human reprogrammed CD8+ T-cells (hrT-cell) on the level of cancer cells and cancer stem cells. Phenotype establishment and qualitative analysis of CD44 APC-Cy™7, CD90 APC, CD117 FITC, CD274 (PD-L1) PE, CD276 FITC, CD279 (PD-1) BV421, EGF (F4/80) Alexa Fluor® 647, Axl BV421 and Sox2 PE expression.

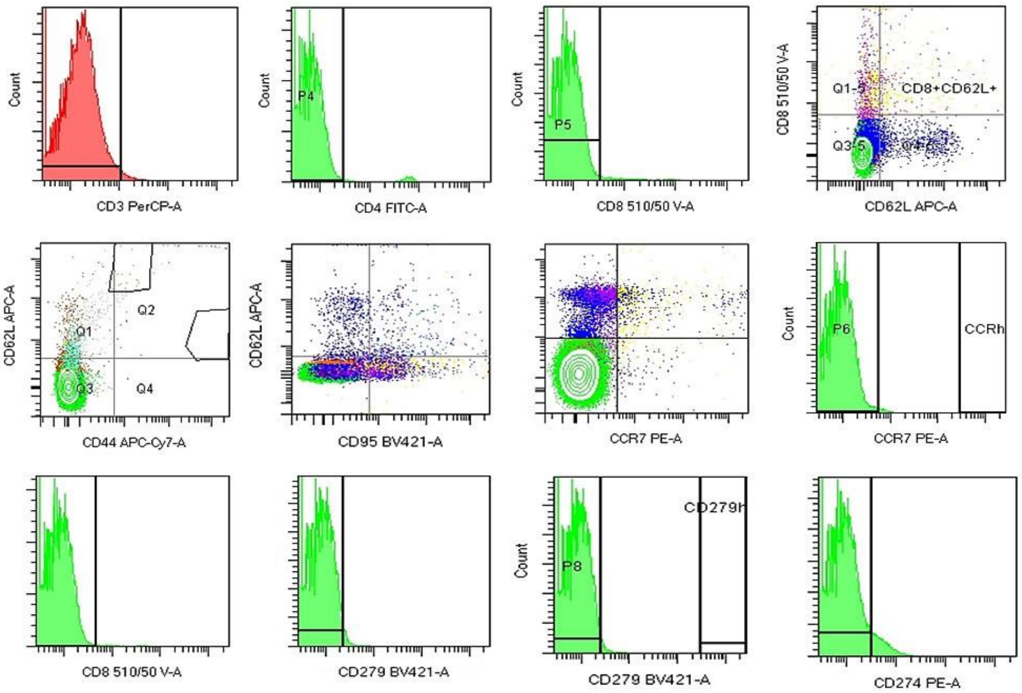

Figure S4. The effect of therapy with human reprogrammed CD8+ T-cells (hrT-cell) on level of T-cells in blood. Phenotype establishment and qualitative analysis of CD3 PerCP, CD4 FITC, CD8 BV510, CD44 APC-Cy™7, CD45RA PerCP-Cy™5.5, CD62L APC, CD95 BV421, CD197 (CCR7) PE, CD274 (PD-L1) PE, and CD279 (PD-1) BV421 expression.
